# Supplementary material for: A Critical View of the Application of the APEX Software (Aqueous Photochemistry of Environmentally-Occurring Xenobiotics) to Predict Photoreaction Kinetics in Surface Freshwaters
Source: Molecules. 2019 Dec 18;25(1):9. doi: 10.3390/molecules25010009 (PMC7017383; doi:10.3390/molecules25010009)
Supplement: Supplementary file 1 [file molecules-25-00009-s001.zip › 3.2.4_gcc-4.4.0.zip!/3.2.4_gcc-4.4.0/3.2.4_gcc-4.4.0/doc/octave/html/interpreter/Geometry.html]

Geometry - Untitled


Next: Signal Processing,
Previous: Interpolation,
Up: Top


---

## 29 Geometry

Much of the geometry code in Octave is based on the Qhull
library^1^.
Some of the documentation for Qhull, particularly for the options that
can be passed to `delaunay`, `voronoi` and `convhull`,
etc., is relevant to Octave users.

- Delaunay Triangulation- Voronoi Diagrams- Convex Hull- Interpolation on Scattered Data

---

#### Footnotes

[1] Barber, C.B., Dobkin, D.P., and Huhdanpaa, H.T.,
"The Quickhull algorithm for convex hulls," ACM Trans. on Mathematical
Software, 22(4):469–483, Dec 1996, http://www.qhull.org

---
